# The Prevalence of Human Papilloma Virus in Squamous Cell Carcinoma of Oral Tongue

**Published:** 2017-04-01

**Authors:** Mohamad Javad Ashraf, Shahla Hosseini, Ahmad Monabati, Behnaz Valibeigi, Bijan Khademi, Elham Abedi, Negar Azarpira

**Affiliations:** 1 *Pathology Department, Shiraz University of Medical Sciences, Shiraz, Iran*; 2 *Otolaryngology Department, Shiraz University of Medical Sciences, Shiraz, Iran*; 3 *Transplant Research Center, Shiraz University of Medical Sciences, Shiraz, Iran*

**Keywords:** Human Papilloma Virus, Squamous Cell Carcinoma, Tongue

## Abstract

**Background and objective:**

Oral tongue Squamous Cell carcinoma (SCC) commonly involves males between the sixth to eighth decades of life. Major risk factors are tobacco usage and alcohol consumption. The increasing number of patients developing oral tongue cancer without these well-known risk factors suggests that a viral infection, such as Human Papillomavirus (HPV), may be responsible for this increase, by acting as an oncogenic agent. This study investigated the prevalence of HPV infection and its clinicopathologic significance in oral tongue SCCs.

**Methods:**

Tissue blocks from a total of 50 cases (patients with oral tongue SCC) and 50 controls (palatine tonsillar tissues with benign diagnosis) were selected. DNA was extracted from tumoral and non-tumoral tissue blocks. Detection of common HPV DNA by nested Polymerase Chain Reaction (PCR), and high-risk genotypes, HPV 16 and HPV 18, by conventional PCR, was achieved and the results correlated with clinicopathological parameters.

**Results:**

Of the 50 patients (18 males and 32 females with a mean age of 57.36±12.18 years, and age range of 27 to 86 years), 7 (14%) had HPV positive results. None of the control group subjects had HPV DNA positive results (P-value of 0.012). The HPV genotype 16/18 was not detected in positive cases. No statistically significant association was found between HPV status and gender, age, tumor grade, tumor stage or lymph node involvement.

**Conclusion:**

Although there was a significantly higher prevalence of HPV in oral tongue SCC, its association with carcinogenesis in this area requires further studies.

## Introduction

Head and Neck Squamous Cell Carcinoma (HNSCC) is the sixth most common malignancy around the world (1,2). Oral tongue SCC comprises about half of oral cavity SCCs, which commonly involves males between the sixth and eighth decade of life (2,3,4). The major traditional risk factors for oral cavity SCC, including that of the tongue, are tobacco and alcohol consumption (2, 4-10). However, despite a decreasing prevalence of these habits, the incidence of tongue and oral cancer, particularly in young adults, is increasing (4, 9). 

Human Papillomavirus (HPV) is considered as an oncogenic virus (4,9) and may be responsible for this increase (4,10). This virus is an important etiologic factor of HNSCC, particularly tonsil and oropharyngeal cancer, yet, its association with oral tongue SCC is ambiguous. The prevalence of HPV in oral tongue SCC is very variable, ranging from 0% to 100%, in different studies (3-5,9,10). This might be due to the sample size, sample type, storage conditions, detection methods, geographic variation and misclassification of the oropharyngeal tongue as an oral cavity subset (5,7,8).

Human Papillomavirus positive tumors are more common in young patients and present small sized tumors with advanced neck involvement. Histologically, these tumors are poorly differentiated and non-keratinized SCC with basaloid features (11,12,13). Human Papillomavirus positive tumors respond well to chemotherapy and radiation, and have a better prognosis and survival than HPV negative tumors (12,14, 15).

There is limited information on the epidemiology of oral tongue SCC and HPV in Iran. In this study, clinical and tumor characteristics, as well as HPV status in oral tongue SCC, in the south of Iran, was assessed.

## Materials and Methods

Fifty histopathologically-confirmed formalin-fixed, paraffin-embedded tissue blocks of oral tongue SCC, product of partial or complete hemiglossectomy, were obtained from the archives of Khalili Hospital Pathology Laboratory, affiliated to Shiraz University of Medical Sciences, Province of Shiraz, Iran, from 2010 to 2015.

Age, gender, tumor size, TNM stage classification (T stands for Tumor, N stands for lymph node & M stands for metastasis) according to the American Joint Committee on Cancer staging (AJCC) 7^th^ Edition, 2010 (8), histological grade according to the World Health Organization (WHO) guidelines and tumor-node-metastases were obtained from the patients' medical charts. Hematoxylin and Eosin (H&E) slides and previous reports were reviewed by two pathologists. Fifty palatine tonsillar tissue blocks of adult patients with benign diagnosis (chronic tonsillitis and follicular hyperplasia) were collected as a control group for comparative analysis. Based on previous studies, in our study, individuals with age of ≤45 years old were considered as young patients (2,4,7).

**Molecular Study**

Six-micrometer sections from all formalin-fixed, paraffin-embedded tissue blocks were prepared for DNA extraction using the Yekta Tajhiz Azma DNA extraction mini kit for tissue (Iran), according to the manufacturer's instructions.

Detection of common HPV DNA by nested PCR and high-risk genotypes, HPV 16 and HPV 18, by conventional PCR, was performed (9). β-actin primers (317bp) were used as the internal control. For each PCR reaction, DNA extracted from a known HPV-positive uterine cervical tissue was used as the positive control. Each amplification reaction was performed in a total volume of 25 µL containing 200 ng of template DNA, 1X PCR buffer, 1.5 mM mgcl2, 200 µM dNTPs, 1 unit taq DNA polymerase (Yekta Tajhiz Azma) and 0.2 µM of each primer (TIB Molbiol, Germany).

The PCR conditions were as follows; initial denaturation at 94 °C for 5 minutes, followed by 40 cycles of denaturation at 94 ^°^C for 45 seconds, annealing at 55^ °^C for 45 seconds, extension at 72 °C for 45 seconds with a final extension at 72 ^°^C for 5 minutes. Amplicons were run on 2.5% agarose gel, followed by Gel Red staining and visualization with transilluminator

**Human Papillomavirus** **Genotyping**

For identification of HPV 16 and HPV 18, conventional PCR was carried out separately on the HPV positive samples (9). The PCR reactions consisted of a total volume of 25 µL and contained 200 ng of template DNA, 1X PCR buffer, 1.5 mM mgCl_2_, 200 µM dNTPs, 1 unit taq DNA polymerase (Yekta Tajhiz Azma) and 0.2 µM of HPV 16 primers or 0.2 µM HPV 18 primers (TIB Molbiol, Germany).

The PCR reaction was performed with initial denaturation at 94 ^°^C for 1-minute, followed by 35 cycles of denaturation at 94 °C for 1 minute, annealing at 58 ^°^C for 2 minutes, extension at 72 ^°^C for 3 minutes, and final extension step at 72 ^°^C for 10 minutes. Amplicons were run on a 2.5% agarose gel, followed by Gel Red staining and visualization with a transilluminator.

**Statistical Analysis**

The correlation between HPV status in oral tongue SCC and clinicopathological parameters was analyzed using Chi-square and Fisher’s exact tests with the SPSS software (version 14). P-values less than 0.05 were considered statistically significant.

## Results

The patients’ age ranged from 27 to 86 years old (mean age of 57.36±12.18 years), and 36% were male and 64% female. Among the controls, 46% were male and 54% female, and age ranged from 29 to 68 years (mean age of 49.72±10.10 years) (P>0.05). Fourteen cases (28%) were aged ≤45 years and were, therefore, considered as young patients (Table 1).

**Table 1:** Correlation of Human Papillomavirus Status with the Characteristics of Oral Tongue Squamous Cell Carcinoma Cases

Characteristics	HPV		P-Value
	Positive (n=7) (%)	Negative (n=43) (%)	
Age			
≤45 years >45 years	2 (14.3%) 5 (13.9%)	12 (85.7%) 31 (86.1%)	1.000
Gender			
Male Female	5 (27.8%) 2 (6.2%)	13 (72.2%) 30 (93.8%)	0.083
Tumor Differentiation			
Well (n= 26) Moderately (n=18) Poorly (n=6)	6 (23.1%) 0 (0%) 1 (16.7%)	20 (76.9%) 18 (100%) 5 (83.3%)	0.092
Tumor stage			
I II III IV	1 (12.5%) 2 (28.6%) 2 (11.1%) 2 (11.8%)	7 (87.5%) 5 (71.4%) 16 (88.9%) 15 (88.2%)	0.755
Lymph node status			
Free Involved	3 (14.3%) 4 (14.8%)	18 (85.7%) 23 (85.2%)	1.000

**Fig 1:**
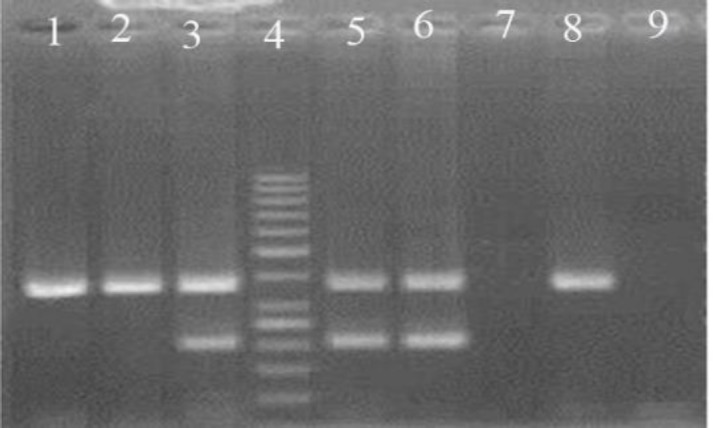
Agarose Stained Gel Representative Polymerase Chain Reaction Products of Tongue Squamous Cell Carcinoma Samples for Human Papillomavirus

Human Papillomavirus DNA was detected in 7 out of 50 (14%) cases with oral tongue SCC (Figure 1).

All tonsillar tissues were negative for HPV genome; this difference was statistically significant (p-value=0.012). The mean age of HPV-positive and HPV-negative patients was 56.71±11.14 and 57.46±11.18 years, respectively (P value of 0.91).

HPV infection was more common in male compared to female patients, although the difference was not statistically significant (27.8% vs. 6.2%, p-value of 0.083). None of the specimens (0%) were positive for HPV 16/18 genotype. Two of 14 (14.3%) young patients (≤45 years old) were HPV positive. In HPV positive cases, most of the patients were more than 45 years old (71.4%) compared with young patients (28.6%), although the difference was not statistically significant (P value of 1).

HPV infection was more common in male compared to female patients, although the difference was not statistically significant (27.8% vs. 6.2%, p-value of 0.083). None of the specimens (0%) were positive for HPV 16/18 genotype. Two of 14 (14.3%) young patients (≤45 years old) were HPV positive. In HPV positive cases, most of the patients were more than 45 years old (71.4%) compared with young patients (28.6%), although the difference was not statistically significant (P value of 1).

The frequency of HPV infection was higher in well differentiated than poorly differentiated tumors, yet, the difference was not statistically significant (23.1% vs. 16.7%, P value of 0.092). None of the moderately differentiated tumors showed HPV positivity.

On the other hand, in HPV positive cases, most of the tumors were well differentiated, yet, the difference was not statistically significant (85.7% vs. 14.3%, P value of 0.092).

Although HPV positivity was more prevalent in stage II tumors (28.6%), no statistically significant differences were observed between HPV status and tumor stage (P value of 0.755).

Also, no statistically significant differences were observed between HPV positivity and lymph node status (P value of 1).

There was no statistically significant association between HPV status and gender, age, tumor grade, tumor stage or lymph node involvement. Correlation of HPV status with the characteristics of cases is shown in Table 1.

## Discussion

Multiple studies have established the causative role of HPV infection in the onset of tonsillar and base of tongue SCC worldwide, yet, its role as a risk factor in oral tongue SCC, has been controversial (5). In our study, HPV DNA was detected in 14% of oral tongue SCC and no high-risk HPV 16/18 genotypes were identified.

Elango et al. from India studied 60 oral tongue SCCs and 46 normal oral mucosal samples. Human Papillomavirus DNA was found in 50% of patients and 67% of the control group, and HPV-16 was detected in 48.3% of the patients (4).

Furthermore, Lee et al. from Korea examined 36 patients with early oral tongue SCC and 25 normal tongue mucosa, using the PCR method. The virus was found in 36% and 4% of tumors and normal oral mucosa, respectively. About 85% of HPV positive cases had HPV-16 genotypes (10).

Paz et al. from the USA studied frozen tissue samples of 167 patients. The HPV DNA was found in 12.8% of tongue SCCs. This study showed that HPV positive patients presented a higher stage of disease compared with HPV negative individuals, yet, there was no significant difference in the 3-year survival rate (16).

Kantola et al. studied 105 cases of oral tongue SCC and found HPV in all patients (17). Moreover, Dahlgren et al. studied 85 oral tongue SCCs and 25 base of tongue SCCs using the PCR method. They found HPV DNA in 2.3% of oral tongue and 40% of base of tongue SCCs (18).

Tsimplaki et al. from Greece evaluated 53 patients with oral tongue SCC. The overall frequency of HPV DNA positivity was 11.3%, while high risk HPV was detected in 7.5% of the samples. They suggested that HPV was more common in female compared to male patients and the infection was not associated with younger age (5).

In India, HPV was more common in males compared to females, with no significant difference in the frequency of HPV infection between younger (≤45 years) and older patients (>45 years) (4). In another study, Liang et al. analyzed fresh-frozen tumor tissues of 51 patients with oral tongue SCC by the PCR assay for the presence of HPV DNA, and found only 1 HPV-positive (HPV-16) tumor (1.96%); this patient was young (4).

It seems that in the Iranian population, SCC is the most common lesion of the tongue (19). The patients' age was between 41 and 60 years and there was a higher frequency of males (53%) compared to females (47%) (19). 

In Mashhad, North East province of Iran, SCC of tongue was the most common oral cancer in young patients (<40 years). Familial history was considered as an important etiological factor and its effect was more significant than tobacco and alcohol consumption. The HPV DNA was not detected in these patients (20). 

Saghravanian et al. studied oral leukoplakia and verrucous **c**arcinoma of oral cavity, and evaluated high risk HPV 16, 18, 31 and 33 by PCR. Human Papillomavirus 16 and 18 were found in 14.3% of verrucous carcinomas, and were located in vestibule of the mandible (21).

Seraj et al. studied 94 paraffin-embedded tissue blocks of patients with oral tongue SCC by the PCR assay for the detection of HPV 16/18. The frequency of HPV 16 and HPV 18 infection was 10.6% and 16%, respectively (7). Human Papillomavirus was more common in males than females, yet there was no association with age (7).

It seems that the prevalence of common and high risk HPV is highly variable in different studies and in different geographic regions. In our study, HPV infection was more common in male compared to female patients. There was no significant difference in mean age of HPV-positive and HPV-negative patients. Also, no significant difference was detected in the frequency of HPV positivity between younger and older patients.

In the current study, the frequency of HPV infection was higher in well differentiated than in poorly differentiated tumors. Other researchers like Elango et al. (4) and Seraj et al. (7) found that HPV was more prevalent in well differentiated than in moderately or poorly differentiated tumors. Also, Dahlgren et al. reported that HPV was more common in undifferentiated compared to well and moderately differentiated tumors.

## Conclusion

 In our study there was a significantly higher prevalence of HPV in oral tongue SCC than in the normal control group, while high risk HPV was not detected. No statistically significant correlation was found between HPV DNA positivity and clinicopathological characteristics. 

The results of the present study did not support the role of HPV infection in carcinogenesis in oral tongue SCC. The possible environmental and host genetic factors must be considered.
